# Discovering Motifs in Ranked Lists of DNA Sequences

**DOI:** 10.1371/journal.pcbi.0030039

**Published:** 2007-03-23

**Authors:** Eran Eden, Doron Lipson, Sivan Yogev, Zohar Yakhini

**Affiliations:** 1 Computer Science Department, Technion, Haifa, Israel; 2 IBM Research Laboratories, Haifa, Israel; 3 Agilent Laboratories, Santa Clara, California, United States of America; Massachusetts Institute of Technology, United States of America

## Abstract

Computational methods for discovery of sequence elements that are enriched in a target set compared with a background set are fundamental in molecular biology research. One example is the discovery of transcription factor binding motifs that are inferred from ChIP–chip (chromatin immuno-precipitation on a microarray) measurements. Several major challenges in sequence motif discovery still require consideration: (i) the need for a principled approach to partitioning the data into target and background sets; (ii) the lack of rigorous models and of an exact *p*-value for measuring motif enrichment; (iii) the need for an appropriate framework for accounting for motif multiplicity; (iv) the tendency, in many of the existing methods, to report presumably significant motifs even when applied to randomly generated data. In this paper we present a statistical framework for discovering enriched sequence elements in ranked lists that resolves these four issues. We demonstrate the implementation of this framework in a software application, termed DRIM (discovery of rank imbalanced motifs), which identifies sequence motifs in lists of ranked DNA sequences. We applied DRIM to ChIP–chip and CpG methylation data and obtained the following results. (i) Identification of 50 novel putative transcription factor (TF) binding sites in yeast ChIP–chip data. The biological function of some of them was further investigated to gain new insights on transcription regulation networks in yeast. For example, our discoveries enable the elucidation of the network of the TF ARO80. Another finding concerns a systematic TF binding enhancement to sequences containing CA repeats. (ii) Discovery of novel motifs in human cancer CpG methylation data. Remarkably, most of these motifs are similar to DNA sequence elements bound by the Polycomb complex that promotes histone methylation. Our findings thus support a model in which histone methylation and CpG methylation are mechanistically linked. Overall, we demonstrate that the statistical framework embodied in the DRIM software tool is highly effective for identifying regulatory sequence elements in a variety of applications ranging from expression and ChIP–chip to CpG methylation data. DRIM is publicly available at http://bioinfo.cs.technion.ac.il/drim.

## Author Summary

A computational problem with many applications in molecular biology is to identify short DNA sequence patterns (motifs) that are significantly overrepresented in a target set of genomic sequences relative to a background set of genomic sequences. One example is a target set that contains DNA sequences to which a specific transcription factor protein was experimentally measured as bound while the background set contains sequences to which the same transcription factor was not bound. Overrepresented sequence motifs in the target set may represent a subsequence that is molecularly recognized by the transcription factor. An inherent limitation of the above formulation of the problem lies in the fact that in many cases data cannot be clearly partitioned into distinct target and background sets in a biologically justified manner. We describe a statistical framework for discovering motifs in a list of genomic sequences that are ranked according to a biological parameter or measurement (e.g., transcription factor to sequence binding measurements). Our approach circumvents the need to partition the data into target and background sets using arbitrarily set parameters. The framework is implemented in a software tool called DRIM. The application of DRIM led to the identification of novel putative transcription factor binding sites in yeast and to the discovery of previously unknown motifs in CpG methylation regions in human cancer cell lines.

## Introduction

### Background

This paper examines the problem of discovering “interesting” sequence motifs in biological sequence data. A widely accepted and more formal definition of this task is: ***given a target set and a background set of sequences (or a background model), identify sequence motifs that are enriched in the target set compared with the background set.***

The purpose of this paper is to extend this formulation and to make it more flexible so as to enable the determination of the target and background set in a data driven manner.

Discovery of sequences or attributes that are enriched in a target set compared with a background set (or model) has become increasingly useful in a wide range of applications in molecular biology research. For example, discovery of DNA sequence motifs that are overabundant in a set of promoter regions of co-expressed genes (determined by clustering of expression data) can suggest an explanation for this co-expression. Another example is the discovery of DNA sequences that are enriched in a set of promoter regions to which a certain transcription factor (TF) binds strongly, inferred from chromatin immuno-precipitation on a microarray (ChIP–chip) [1] measurements. The same principle may be extended to many other applications such as discovery of genomic elements enriched in a set of highly methylated CpG island sequences [2].

Due to its importance, this task of discovering enriched DNA subsequences and capturing their corresponding motif profile has gained much attention in the literature. Any approach to motif discovery must address several fundamental issues. The first issue is the way by which motifs are represented. There are several strategies for motif representation: using a k-mer of IUPAC symbols where each symbol represents a fixed set of possible nucleotides at a single position (examples of methods that use this representation include REDUCE [3], YMF [4,5], ANN-SPEC [6], and a hypergeometric-based method [7]) or using a position weight matrix (PWM), which specifies the probability of observing each nucleotide at each motif position (for example MEME [8], BioProspector [9], MotifBooster [10], DME-X [11], and AlignACE [12]). Both representations assume base position independence. Alternatively, higher order representations that capture positional dependencies have been proposed (e.g., HMM and Bayesian networks motif representations [13]). While these representations circumvent the position independence assumption, they are more vulnerable to overfitting and lack of data for determining model parameters. The method described in this paper uses the k-mer model with symbols above IUPAC.

The second issue is devising a motif scoring scheme. Many strategies for scoring motifs have been suggested in the literature. One simple yet powerful approach uses the hypergeometric distribution for identifying enriched motif kernels in a set of sequences and then expanding these motifs using an EM algorithm [7]. The framework described in this paper is a natural extension of the approach of [7]. YMF [4,5] is an exhaustive search algorithm which associates each motif with a *z*-score. AlignACE [12] uses a Gibbs sampling algorithm for finding global sequence alignments and produces a MAP score. This score is an internal metric used to determine the significance of an alignment. MEME [8] uses an expectation maximization strategy and outputs the log-likelihood and relative entropy associated with each motif.

Once a scoring scheme is devised, a defined motif search space is scanned (either heuristically or exhaustively) and motifs with significantly high scores are identified. To determine the statistical significance of the obtained scores, many methods resort to simulations or ad hoc thresholds. Several excellent reviews narrate the different strategies for motif detection and use quantitative benchmarking to compare their performance [14–18]. A related aspect of motif discovery, which is outside the scope of this paper, focuses on properties of clusters and modules of TF binding sites (TFBS). Examples of approaches that search for combinatorial patterns and modules underlying TF binding and gene expression include [19–23].

### Open Challenges in Motif Discovery

One issue of motif discovery that is often overlooked concerns the partition of the input set of sequences into *target* and *background* sets. Many methods rely on the user to provide these two sets and search for motifs that are overabundant in the former set compared with the latter. The question of how to partition the data into target and background sets is left to the user. However, the boundary between the sets is often unclear and the exact choice of sequences in each set arbitrary. For example, suppose that one wishes to identify motifs within promoter sequences that constitute putative TFBS. An obvious strategy would be to partition the set of promoter sequences into target and background sets according to the TF binding signal (as measured by ChIP–chip experiments). The two sets would contain the sequences to which the TF binds “strongly” and “weakly,” respectively. A motif detection algorithm could then be applied to find motifs that are overabundant in the target set compared with the background set. In this scenario, the positioning of the cutoff between the strong and weak binding signal is somewhat arbitrary. Obviously, the final outcome of the motif identification process can be highly dependent on this choice of cutoff. A stringent cutoff will result in the exclusion of informative sequences from the target set while a promiscuous cutoff will cause inclusion of nonrelevant sequences—both extremes hinder the accuracy of motif prediction. This example demonstrates a fundamental difficulty in partitioning most types of data. Several methods attempt to circumvent this hurdle. For example, REDUCE [3] uses a regression model on the entire set of sequences. However, it is difficult to justify this model in the context of multiple motif occurrence (as explained below). In other work, a variant of the Kolmogorov-Smirnov test was used for motif discovery [24]. This approach successfully circumvents arbitrary data partition. However, it has other limitations such as the failure to address multiple motif occurrences in a single promoter, and the lack of an exact characterization of the null distribution. Overall, the following four major challenges in motif discovery still require consideration: (c1) the cutoff used to partition data into a target set and background set of sequences is often chosen arbitrarily; (c2) lack of an exact statistical score and *p*-value for motif enrichment. Current methods typically use arbitrarily set thresholds or simulations, which are inherently limited in precision and costly in terms of running time; (c3) a need for an appropriate framework that accounts for multiple motif occurrences in a single promoter. For example, how should one quantify the significance of a single motif occurrence in a promoter against two motif occurrences in a promoter? Linear models [3] assume that the weight of the latter is double that of the former. However, it is difficult to justify this approach since biological systems do not necessarily operate in such a linear fashion. Another issue related to motif multiplicity is low complexity or repetitive regions. These regions often contain multiple copies of degenerate motifs (e.g., CA repeats). Since the nucleotide frequency underlying these regions substantially deviates from the standard background frequency, they often cause false-motif discoveries. Consequently, most methods mask these regions in the preprocessing stage and thereby lose vital information that might reside therein; (c4) criticism has been made over the fact that motif discovery methods tend to report presumably significant motifs even when applied on randomly generated data [25]. These motifs are clear cases of false positives and should be avoided.

### Data Lends Itself to Ranking in a Natural Manner

In this paper we describe a novel method that attempts to solve the above-mentioned four challenges in a principled manner. It exploits the following observation: data often lends itself to ranking in a natural manner, e.g., ranking sequences according to TF binding signal: ranking according to CpG methylation signal, ranking according to distance in expression space from a set of co-expressed genes, ranking according to differential expression, etc. We exploit this inherent ranking property of biological data in order to circumvent the need for an arbitrary and difficult-to-justify data partition. Consequently, we propose the following formulation of the motif finding task: ***given a list of ranked sequences, identify motifs that are overabundant at either end of the list.***

Our solution employs a statistical score termed mHG (minimal hypergeometric) [26]. It is related to the concept of *rank-imbalanced motifs,* which are sequence motifs that tend to appear at either end of a ranked sequence list. In previous work [26], the authors used mHG to identify sequence motifs in expression data. We use this simple yet powerful approach as the starting point for our study.

### Overview

The rest of this paper is divided into two main parts, each of which is self-contained: in the Results we briefly outline our method and describe new biological findings that were obtained by applying this method to biological data. We address challenge (c4) by testing the algorithm on randomly ranked real genomic sequences. In the Methods, we describe the mHG probabilistic and algorithmic framework and explain how we deal with challenges (c1)–(c3).

## Results

### Statistics and Algorithms in a Nutshell

**Figure 1:**
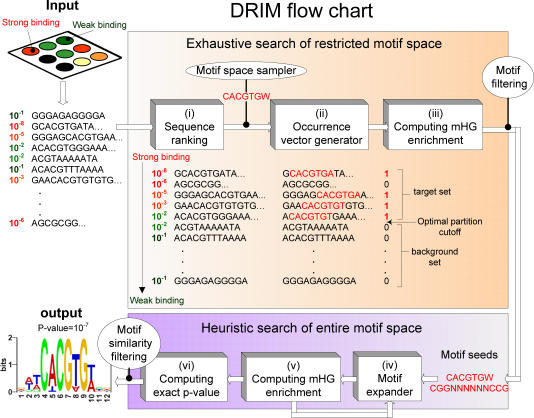
DRIM Flow Chart DRIM receives a list of DNA sequences as input and a criterion by which the sequences should be ranked, for example, TF binding signals as measured by ChIP ChIP–chip: (i) The sequences are ranked according to the criterion. (ii) A “blind search” is performed over all the motifs that reside in the restricted motif space (in this study the restricted motif space contains ∼100,000 motifs, see Methods, The DRIM software). For each motif an occurrence vector is generated. Each position in the vector is the number of motif occurrences in the corresponding sequence, (the figure shows the vector for the motif CACGTGW). (iii) The motif significance is computed using the mHG scheme, and the optimal partition into target and background sets in terms of motif enrichment is identified. The promising motif seeds are passed as input to the heuristic motif search model and the rest are filtered out. (iv,v) The motif seeds are expanded in an iterative manner (the mHG is computed in each lap), until a local optimum motif is found. (vi) The exact mHG *p*-value of the motif is computed. If it has a *p*-value < 10^−3^, then it is predicted as a true motif (the choice of this threshold is explained in Results, Proof of principle). The output of the system is the motif representation above IUPAC, its PSSM, mHG *p*-value, and optimal set partition cutoff.

Based on the mHG framework, we developed a software tool termed DRIM (*discovery of rank imbalanced motifs*) for motif identification in DNA sequences. A flow chart of DRIM is provided in Figure 1. The formal introduction and details of the mHG statistics are given in Methods. However, to facilitate the explanation and interpretation of our biological results, we begin with a brief description of the method.

Suppose we are given a set of DNA sequences and some measured signal associated with each sequence. We rank the sequences according to the signal. Now, given a sequence motif, we wish to assess whether that motif tends to appear more often at the “top” of a list compared with the “remainder” of the list. The mHG score captures this type of motif significance. More precisely, the mHG score reflects the surprise of seeing the observed density of motif occurrences at the top of the list compared with the rest of the list under the null assumption that all configurations of motif occurrences in the list are equiprobable. A unique feature of the mHG statistics is that the cutoff between the top and the rest of the list is chosen in a data-driven manner so as to maximize the motif enrichment. This is done by computing the motif enrichment over all possible set partitions and identifying the cutoff at which maximal statistical significance is observed.

The search for this optimal cutoff introduces a multiple testing problem. To solve this without resorting to multiple testing corrections, which diminish the score's sensitivity, we provide a novel algorithm for computing the exact *p*-value of mHG scores (see Methods, Calculating the *p*-value of the mHG score). This eliminates the need to resort to simulations or exhaustively calculated tables.

Our method also includes a new approach to modeling motif multiplicity by incorporating a multidimensional hypergeometric framework (see Methods, Multidimensional mHG score). Unlike some models, which assume linearity (e.g., that two binding motifs have twice the binding capacity as one motif), our model does not make such pre-assumptions. Instead, the degree of surprise is adjusted for each motif according to its own occurrence multiplicity distribution.

DRIM scans through a motif space, computes the mHG *p*-value of these motifs and reports the significant ones (see Methods, The DRIM software).

### Proof of Principle

We begin by testing our method on synthetically generated clear-cut positive and negative control cases. We do this to verify that DRIM accurately identifies motifs in well-characterized and experimentally verified examples and at the same time avoids false identification of motifs in randomly ordered genomic sequences. The latter objective is of particular importance since the issue of false identification has been mentioned as one of the main shortcomings of motif discovery approaches. For example, in a previous study, six different motif discovery applications were used to search for TFBS motifs [25]. Each of the programs attempted to measure the significance of its results using one or more enrichment scores. The authors report that the applications outputted high-scoring motifs even when applied to random selections of intergenic regions. A different paper reports clusters of genes whose expression patterns correlate to the expression of a particular TF [27]. These clusters were then analyzed for enriched motifs. Again, the authors report that random sets, with sizes matching those of the real clusters, contained a large number of motifs with significant scores.

To test our method's false-prediction rate, we performed the following negative control experiment: five different random permutations of ChIP–chip data were generated by randomly selecting 400 promoters and randomly permuting their ranks. DRIM was then applied to these ranked lists and scanned more than 100,000 different motifs in each one. None of the motifs that were scanned had a significant corrected mHG *p*-value <10^−3^. Note that to get the corrected *p*-values, two levels of multiple test corrections are performed: correcting for the number motifs that are tested; and correcting for multiple cutoffs that are tested as part of the mHG optimization process.

How do the *p*-values of random motifs compare with those of true biological motifs? To test this, we chose five TFs (BAS1, GAL4, CBF1, INO2, and LEU3) whose motif binding sites are well-characterized and experimentally verified. We applied DRIM to the ChIP–chip data of these TFs as reported in [25]. In all instances, the true motifs were identified with corrected *p*-values of 10^−6^, 10^−9^, 10^−76^, 10^−18^, and 10^−8^, respectively. A comparison of the *p*-value distribution of the motifs in the randomly ordered sequences with that of the verified TFBS motifs is given in Figure S3. In all instances the true TFBS motifs were predicted with *p*-values that were several orders of magnitude more significant than the best *p*-value of a motif in the randomly permuted data. This indicates that the enrichment signals of true TFBS, as captured by the mHG *p*-value, are clearly distinct from the signals we expect to find in random rankings of genomic sequences.

### TFBS Prediction Using ChIP–chip

To further test the effectiveness of our method, we used it for identification of TFBS in yeast by applying it to the Harbison and Lee–filtered ChIP–chip datasets [25,28], containing measurements of 207 TF binding experiments in several conditions (for details regarding dataset-filtering see Methods). Interestingly, we observed that in many of these datasets longer intergenic regions are biased toward stronger TF binding. We elaborate on this sequence length bias in the Methods section and in Figure S1.

In each of the ChIP–chip experiments, we ranked the intergenic regions according to the TF binding signal (we use the *p*-value of enrichment for the sequence represented on the array). This was used as input for DRIM, which then searched for motifs that tend to appear densely at the top of the ranked lists. If such a motif does exist, with a *p*-value less than 10^−3^, then we hypothesize that it is biologically significant and that it contributes to the TF's binding, either directly or indirectly.

**Figure 2:**
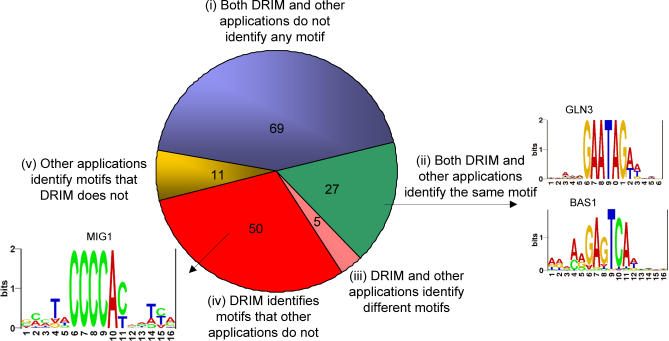
Comparison between Predictions of DRIM and Published Predictions of Six Other Methods and Conservation Data as Reported in [25] Overall, out of 162 unique TFs, DRIM identified significant motifs for 82 TFs with *p*-value <10^−3^. Out of the 162 TFs, DRIM and the other applications agree on 96 TFs: 27 TFs for which a similar motif was found and 69 TFs for which no significant motifs were found. There are five TFs for which the motifs predicted by DRIM and other applications differ; 11 for which the other applications identified motifs that DRIM did not; and 50 for which DRIM identified a motif that the other applications did not (for details see Tables S2 and S3). Sequence logos were generated using the *RNA Structure Logo* software [56].

The results on the Harbison filtered dataset are summarized in Table S2. A TF was assigned a motif if such was found in at least one condition. We compared the DRIM predictions with previously reported TFBS discoveries in ChIP–chip that incorporated predictions of six other motif discovery methods and conservation data [25]. The results of this comparison are summarized in Figure 2.

**Figure 3:**
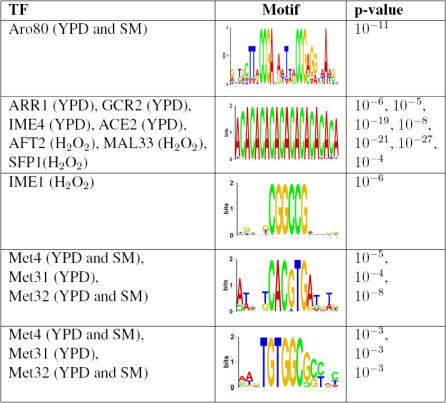
Examples of TFs for Which DRIM Identifies Novel Motifs We further investigated these motifs and show evidence of their biological function. YPD, H_2_O_2_, and SM denote the ChIP–chip experimental conditions [25] in which the motifs were identified.

Overall, DRIM identified 50 motifs that were not picked up by the six other methods as reported in [25]. We further investigated these putative TFBS for additional evidence that they are biologically meaningful. First, we found that seven of them (ASH1, GCR1, HAP2, MET31, MIG1, RIM101, and RTG3) are in agreement with previously published results that are based on experimental techniques other than ChIP–chip. Second, we compared them with a list of conserved regulatory sites in yeast that was recently inferred using conservation-based algorithms [29]. Ten of our putative TFBS match these conserved sites (ARG81, ARO80, ASH1, CRZ1, DAL81, HAP2, IME1, MET31, MIG1, and RTG3). Taken together, these findings provide a strong indication that at least some of the new motifs identified by DRIM are true biological signals. In the following subsections, we focus on a few of these putative TFBS (see Figure 3) and present additional evidence that supports their biological role. We use these findings to discover new interactions in the yeast genetic regulatory network.

#### Aro80 transcription regulatory network.

The Aro80 TF regulates the utilization of secondary nitrogen sources such as aromatic amino acids, as part of the Ehrlich pathway [30]. In particular, it is involved in the regulation of 2-phenylethanol, a compound with a rose-like odor, which is the most-used fragrance in the perfume and cosmetics industry [31]. Due to its commercial potential, the optimized production of this substance has received much attention [31].

We identified the remarkably large motif, WWNCCGANRNWNNCCGNRRNNW, in Aro80 rich media ChIP–chip data [25] with *p*-value < 10^−11^ (see Figure 3). We refer to this putative binding site as BS_Aro80_. Furthermore, we discovered the same motif in two other independent sources of data: Aro80 rich media experiment in the Lee filtered dataset and Aro80 SM condition (amino acid starvation), both with *p*-value < 10^−6^. Only seven copies of this motif occurred in the entire yeast genome. These seven copies are distributed among four promoters, three of which have two copies of BS_Aro80_ each. This unusual motif distribution is combinatorially surprising and therefore suggests biological significance. We note that BS_Aro8_ shares some similarity with a previously reported Aro80 motif [29,32]. However, the sequence of BS_Aro8_ provides new insights into the mechanism of the yeast Ehrlich pathway that cannot be explained by the previously described motif. (i) It was previously shown that Aro80 enhances the transcription of Aro9 and Aro10 [30,32]. We found BS_Aro80_ in the promoters of both genes—two copies in each promoter. (ii) Interestingly, BS_Aro80_ appears in the promoter of the gene coding to the Aro80 protein. Since the BS_Aro8_ motif appears only in four promoters in the entire genome, it is highly unlikely that this occurred by chance. We therefore hypothesize that Aro80 self-regulates its own transcription by directly binding to its own promoter. (iii) The fourth promoter (when ranking according to Aro80 rich media ChIP–chip data [25]) contains two BS_Aro80_ elements, one on the sense and the other on the anti-sense. This configuration is shared by two divergently transcribed genes, NAF1 and Esbp6. The latter gene was previously shown to have increased transcription in the presence of phenylalanine as sole nitrogen source [30], suggesting it may play a role in the Ehrlich pathway. Esbp6 is a monocarboxylate permease and might be involved in the transfer of substrates of the Ehrlich pathway across the plasma membrane. (iv) We analyzed the conservation of BS_Aro8_ in four yeast strains and found all seven of its copies to be conserved in the different strains. (v) Aro80 belongs to the *Zn*_2_ *Cys*_6_ family of TFs that are known to bind CCG elements separated by a spacing. Indeed, in addition to other conserved nucleotides, the motif contains CCG gapped tri-nucleotides. (vi) In a previous study, in order to identify *cis*-acting sequences involved in Aro9 induction, a series of deletions were produced in the Aro9 promoter region, and the expression of a reporter gene was monitored [32]. The authors concluded that the sequence CCGN^7^CCGN^7^CCGN^7^CCG in the Aro9 promoter is responsible for Aro80 binding. We note, however, that the changes in expression caused by the mutations can be interpreted differently, and in fact they are even more consistent with our BS_Aro80_ motif. Deletions or mutations that simultaneously altered all motif copies in the promoter dramatically reduced expression, while those which altered only some of the copies caused a more mild decrease. Other deletions that did not affect any BS_Aro80_ motif did not affect the expression at all. A detailed analysis of the BS_Aro80_ element with respect to these mutagenesis studies is given in Figure S4.

**Figure 4:**
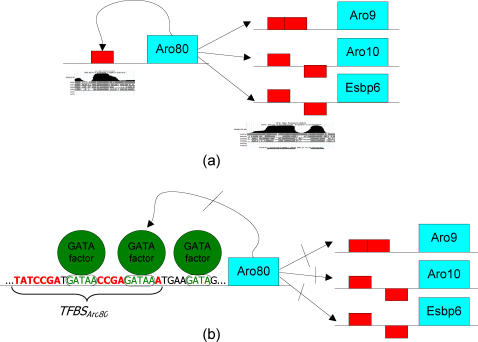
The Hypothetical Regulatory Network of Aro80 Copies of the BS*_Aro_*_80_ motif (on the sense and antisense) are shown as rectangles on the promoter regions. (A) BS*_Aro_*_80_ is conserved in four strains of yeast as shown using the University of California Santa Cruz browser conservation plots. Aro80 regulates the utilization of secondary nitrogen sources such as aromatic amino acids by binding genes that participate in the catabolism of aromatic amino acids. We hypothesize that it also binds to its own promoter region and introduces a positive feedback self loop. (B) Part of the Aro80 promoter sequence is shown with bases of the BS*_Aro_*_80_ motif colored in red. Interestingly, there are three GATA binding sites that are adjacent to the BS*_Aro_*_80_ motif (bases colored in green). These sites bind GATA factors that are known to play a role in nitrogen catabolite repression. We hypothesize that they are also involved in the repression of Aro80 expression by physically binding to the region near BS*_Aro_*_80_, thus making it inaccessible to Aro80 binding. This in turn breaks the positive feedback loop and represses the expression of Aro80 itself and other Aro80 regulated genes.

A putative transcription network of Aro80 that incorporates these findings is shown in Figure 4. Note that GATA binding sites are found adjacent to the BS_Aro80_ motif. We further discuss the potential role of these motifs in the Discussion.

The predicted motif BS_Aro80_ exemplifies the usefulness of the mHG flexible cutoff. Our process partitioned the data into a target set containing the top first four promoters (the only promoters in the genome in which the motif resides) and a background set containing the rest of the promoters. Other methods that used a fixed binding signal cutoff (*p*-value < 10^−3^) for partitioning the data included 16 other promoters in the target set, in addition to the four promoters in which BS_Aro80_ appears. Consequentially, the signal-to-noise ratio decreases, which might explain why other methods did not identify the BS_Aro80_ element.

Taken together, our results suggest the predicted BS_Aro80_ motif is indeed an Aro80 binding site.

#### CA repeats are correlated with TF binding.

We identified a bi-nucleotide CA repeat motif with variable length ranging from six to 62 in the Harbison filtered dataset. The CA repeat motif was found to be highly enriched for seven TFs: ARR1, GCR2, IME4, and ACE2 in rich media condition and AFT2, MAL33, and SFP1 in H_2_O_2_Hi condition. Furthermore, for two of these TFs (GCR2, IME4), we rediscovered the same CA repeat motif in the Lee filtered dataset. In other words, for the specified TFs, we identify a highly significant correlation between a sequence's capacity to bind the TF and the presence of a CA repeat in the sequence. This type of low complexity motifs are often filtered by current methods. One exception is a recent work in which a CACACACACAC sequence was found to be enriched in Rap1 experiments [33].

It has been previously hypothesized that CA repeats might have a functional role in TF binding [34]. It was proposed that CA repeats, which are often conserved in evolutionary distant organisms, are likely to impose a unique DNA tertiary structure that aids in the identification of other specific regulatory elements [34]. Our findings constitute concrete evidence to this phenomena in seven (of 82) different TFs. They are also in agreement with another study in which CA repeat–containing sequences in the human gamma-globin gene promoter required for efficient transcription were identified using in vitro site-directed mutagenesis [35]. Taken together, our findings and other observations suggest CA repeats play a role in the DNA binding of some TFs.

#### Detection of indirect TF–DNA binding using ChIP–chip.

IME1 is a TF that activates transcription of early meiotic genes. We identified a motif, CGGCCG, with *p*-value < 10^−11^ that is enriched in the sequences to which IME1 binds in H_2_O_2_ condition. Although this motif was not identified by other methods as reported in [25], we found evidence that suggests it is biologically meaningful. First, we note that this motif is a perfect palindrome, which is often characteristic of TF binding sites. Second, the same motif was identified as evolutionarily conserved in IME1-bound sequences as inferred from ChIP–chip data [29]. Third, IME1 interacts with Ume6, also a transcriptional regulator of early meiotic genes, which was previously shown to bind the same DNA motif, CGGCCG [36]. We conclude that the IME1-discovered motif is likely due to the following scenario: IME1 binds to Ume6, which binds to CGGCCG sequences on the DNA. The cross linking in the ChIP–chip protocol fixes these bindings, and the immunoprecipitation of IME1 actually precipitates the entire complex. We therefore get enriched CGGCCG sequences in IME1 experiments due to indirect binding to this DNA motif.

In another example, we identified the same two distinct motifs, *M*_1_ = TGTGGCSS and *M*_2_ = CACGTG, in rich media ChIP–chip experiments of three different TFs: Met4, Met31, and Met32. Furthermore, we rediscovered the same motifs in other experimental conditions of the same TFs. Met4, Met31, and Met32 are three factors involved in the sulfur amino acid pathway, and the fact that the same two motifs were independently predicted for each of the TFs is unlikely to occur by chance, suggesting the predictions are biologically meaningful. In a previous work it was shown that Met4 is tethered to the DNA sequence AAACTGTG via two alternative complexes, Met4-Met28-Met31 and Met4-Met28-Met32 (the binding is thought to occur via Met31/32) [37]. This sequence partially overlaps motif *M*_1_. Furthermore, the complex Met4-Met28-Cbf1 was shown to bind motif *M*_2_ [38]. Previous findings are summarized in Figure S5A. The above explains why we predict *M*_1_ for Met4 and *M*_2_ for Met31/32. However, it does not explain why we also predict *M*_2_ for Met4 and *M*_1_ for Met31/32. The most likely explanation for this is the existence of a direct interaction between the two complexes Met4-Met28-Cbf1 and Met4-Met28-Met31/32. If such an interaction exists, then the cross linking would fix the two complexes and cause the immunoprecipitation of Met4, Met31, and Met32 to precipitate the same set of sequences, thus causing the same motifs to appear in the experiments of all three TFs, which is exactly what DRIM identifies. This point is illustrated in Figure S5B. The idea of direct interaction between the two complexes is also in agreement with previous results [37].

Overall, the results shown in this subsection demonstrate that DRIM is able to identify previously ignored subtle signals in ChIP–chip data that stem from indirect bindings of factors to DNA. This type of information can be useful for inferring novel protein–protein interactions.

#### Condition-dependent motifs.

A comparison was made between the predicted motifs of the same TF in different experimental conditions (see Table S2). These seem to fall into two main categories: (i) motifs whose enrichment is condition-dependent, and (ii) motifs whose enrichment is condition-independent, suggesting the TF is bound to the DNA regardless of condition. In the latter, although the same motif was predicted in different conditions, the motif enrichment varied considerably. For instance, the GAL4 binding site CGGN^11^CCG, previously reported in [1] and other literature, was predicted in both YPD and galactose conditions. However, the enrichment varied considerably with *p*-values 10^−7^ and 10^−11^, respectively. This several-fold difference in enrichment is consistent with what is known about the role of GAL4 in galactose metabolism. It suggests that GAL4 has a preference to bind CGGN^11^CCG DNA regardless of condition. However, in the presence of galactose and absence of glucose, this preference becomes much more significant. Another example of a condition-invariant motif whose binding strength is subject to experimental condition is that of the Aro80 TF. This demonstrates that DRIM can be used not only to identify binding sites but also to distinguish between different modes of TF binding.

### Motif Discovery in Human Methylated CpG Islands

To examine our method's ability to predict sequence motifs that stem from data other than TF binding, DRIM was applied to a dataset containing the human cancer cell line–methylated CpG islands (for dataset details, see Methods) to seek for motifs that are enriched in hypermethylated regions. The promoters were ranked according to methylation signal, with hypermethylated promoters at the top. Note that different replicates of the same cell line may yield different ranking of the promoters.

DRIM identified significantly enriched motifs in each of the four cancer cell lines. Table 1 shows all the motifs that were independently discovered in at least two different replicates of the same experiment or that are in agreement with previous work [2]. Overall, DRIM discovered 13 motifs: ten novel motifs and three that have been previously predicted in hypermethylated CpG island promoters in the same cancer cell lines [2]. Some of these motifs have also been independently identified in methylated CpG regions of other cell lines [39,40].

**Table 1:**
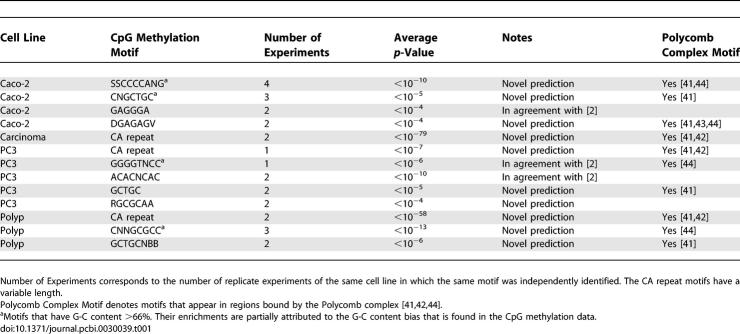
Enriched Motifs Associated with CpG Methylation in Four Human Cancer Cell Lines and Comparison to Motifs in Regions Bound by the Polycomb Complex

Interestingly, nine of the novel ten motifs were independently identified in DNA regions to which the proteins of the Polycomb complex bind [41–43]. The Polycomb complex is involved in gene repression through epigenetic silencing and chromatin remodeling, a process that involves histone methylation. The fact that these two distinct key epigenetic repression systems, namely histone methylation and CpG methylation, bind to regions that share a similar set of sequence motifs suggests they are linked. To further establish this link we applied DRIM to Polycomb complex bound promoters in human embryonic fibroblasts [44]. We found four motifs that are similar to the CpG methylation motifs (Table 1). Our findings are consistent with a recent paper that showed that the EZH2 Polycomb protein binds methyltransferases via the Polycomb complex [45].

Most of the motifs we found are similar across more than one type of cancer cell line, e.g., variants of the GCTGCT motif appear in Caco-2, PC3, and Polyp1 cancer cell lines. This suggests that the same DNA binding factors are involved in CpG methylation of different types of cancers. It is also important to note that some of the motifs we discovered are G–C rich. The enrichment of these motifs may be partially attributed to the G–C content bias that is found in CpG methylation data.

The DRIM motif identification process can be used not only to identify novel motifs but also to partition the data in a biologically meaningful manner. In [2] the authors used a fixed threshold on the methylation signal (*p*-value < 0.001 ) to partition the dataset. Consequently, they identified 135 hypermethylated promoters. A data-driven partition would be to use the threshold that yielded the maximal motif enrichment. For example, in the Caco2 cell line, we identified the same motif as in the previous work [2]. However, the motif maximal enrichment was found in the top 209 promoters (an increase of 54% in target set size).

### Motif Discovery in Human ChIP–chip Data

Human TFBS tend to be longer and “fuzzier” than TFBS of lower eukaryotes, and it is important to evaluate our method's performance on such motifs. To this end, we applied DRIM to the ChIP–chip experiments of HNF1α, HNF4α, HNF6 in liver and pancreas islets [46], as well as to that of CREB [47]. For each of the TFs, we generated a list of sequences containing 1,000 bases upstream and 300 downstream of the transcription start site (TSS). We ranked the list according to the TF ChIP–chip signal and used it as input to DRIM. DRIM successfully detected the TFBS of these TFs that are reported in TRANSFAC with extremely significant *p*-values: HNF1α liver—GTTAMWNATT (*p* = 10^−8^), HNF4α Islets—SCGGAAR (*p* = 10^−53^), HNF6 Liver—ATCRAT (*p* = 10^−57^), and HNF6 Islets—ATCRAT (*p* = 10^−61^). In the CREB experiments we identified the palindromic motif TGACGTCA (*p* = 10^−16^), which is known to bind CREB [47].

### Comparison with Other Methods

Three properties of the mHG enrichment score embodied in DRIM offer advantages over other motif discovery methods: the dynamic cutoff, the rigorous control over false positives, and the motif multiplicity model.

#### Dynamic versus rigid cutoffs.

Most methods use an arbitrary cutoff for set partition. For example, in previous work [25] the authors use a cutoff of *p*-value < 10^−3^ on the ChIP–chip signal in order to define the target set for motif searching. In contrast, the mHG score uses a data-driven flexible cutoff and chooses the set partition that maximizes the motif enrichment.

**Figure 5:**
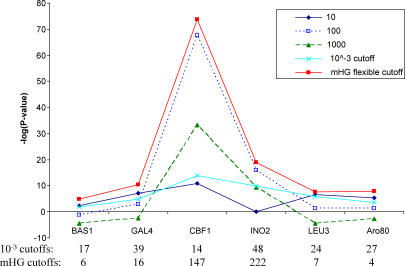
Comparison between HG and mHG Enrichment The mHG and HG methods were applied to ChIP–chip data of six TFs. The sequences were ranked according to the ChIP–chip binding signal, and the enrichment of the correct binding motif was recorded using mHG and HG with fixed target sets containing the top 10, 100, and 1,000 sequences as well as all sequences with ChIP–chip signal <10^−3^. All scores were corrected for multiple motif testing. The mHG score is also corrected for the multiple cutoff testing. The 10^−3^ and mHG cutoffs for each experiment are shown. It can be seen that the two cutoffs are significantly different and that for all the tested TFs mHG produces better results than HG in terms of enrichment of the true motif.

To more systematically investigate the advantages of using a flexible cutoff, we compared mHG with fixed set partition HG [7] by disabling the flexible cutoff feature in DRIM. The comparison was performed on ChIP–chip data of TFs whose motif binding sites are well-characterized as well as on the Aro80 binding site we identified. For each TF, we ranked the sequences according to the ChIP–chip binding signal, generated the motif occurrence vector, and computed its HG enrichment using fixed target sets containing the top 10, 100, and 1,000 sequences as well as all sequences with ChIP–chip signal <10^−3^. The results are summarized in Figure 5. We note that all of the scores are corrected for multiple-motif testing. The mHG score is also corrected for the multiple-cutoff testing. The mHG method yields superior results in all six cases.

We performed additional comparisons of the mHG versus the HG methods by applying both methods to simulations of motif occurrence vectors (see Text S7 and Figure S6). In these simulations mHG showed significantly better performance than HG.

**Figure 6:**
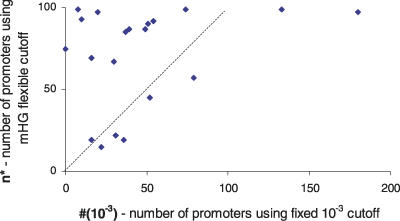
Comparison of the Target Sets Sizes as Determined by the Fixed versus the mHG Flexible Cutoffs Each dot represents a ChIP–chip experiment where the *x* and *y* coordinates are the number of promoters with *p* < 10^−^ (standard cutoff) and the number of promoters as determined by the mHG cutoff, respectively. The dotted line is *x* = *y*. TF names are given in Table S4.

To further investigate the issue of setting a cutoff, we compare, for a given TF and condition in the ChIP–chip dataset, the number of promoters for which the binding signal <10^−3^ (denoted #(10^−3^)) with the number of promoters at which mHG was attained (denoted *n* ^*^). For 82 experiments, #(10^−3^) ≤ 4 and for 46 of these #(10^−3^) = 0. In these cases a 10^−3^ fixed cutoff reduces the size of the target set and limits the usability of any discovery algorithm. In Figure 6 we compare #(10^−3^) and *n* ^*^ for some of the cases at which a motif was found by mHG. Note that in a significant number of cases the mHG score identified a significantly enriched motif even when #(10^−3^) was very low. One extreme case is the TF SOK2 in YPD condition for which#(10^−3^) = 0, yet mHG found a significantly enriched motif.

#### Controlling false positives.

The second advantageous property of the mHG score is its ability to rigorously control false positives, due to calculation of an exact *p*-value. This attribute is best demonstrated by comparing the performance of DRIM versus other motif-finding tools on negative controls, that is, datasets whose original ranking was randomly permuted. It is clear that in these cases we should not find significantly enriched motifs. To this end we used the same benchmark on which DRIM was tested (see Results, Proof of principle). Using the same five random permutations of ChIP–chip data, we applied the algorithms AlignACE [12], MEME [8], and MDscan [33] on each of the random sets. Both AlignACE and MEME reported significant motifs with many A's, probably due to the existence of polyA tails in the intergenic regions. MDscan was used with a precompiled background from yeast intergenic regions, and therefore it did not report the polyA motifs, yet it did report motifs including repeats of TA, probably as a result of TATA boxes. In comparison, DRIM did not identify any significant motifs in any of the random sets.

#### Binary versus multidimensional enrichment.

The third advantageous property is the extension of the binary enrichment analysis to the multidimensional enrichment analysis (see Methods, Multidimensional mHG score). The latter forms the basis for dealing with motif multiplicity in a data-driven manner. To test this property, we compared DRIM, which uses the multi-mHG framework, with a restricted version of DRIM, which uses the standard binary enrichment framework. Out of 31 binding motifs identified by DRIM that were also identified in other literature, the restricted version was able to identify only 23. Furthermore, in some instances, both methods were able to identify the correct motif site; however, the motif significance using the multi-mHG framework was several fold more significant without incurring additional false predictions.

## Discussion

In this paper we examine the problem of discovering “interesting” motif sequences in biological sequence data. While this problem has often been regarded as tantamount to discovering enriched motifs in a target set versus a background set, we point out an inherent limitation to this formulation of the problem. Specifically, in most cases, biological measurement data does not lend itself to a single, well-substantiated partition into target and background sets. It does, however, lend itself to ranking in a natural manner. Our approach exploits this natural ranking and attempts to solve challenges (c1)–(c4) (see Introduction, Open challenges in motif discovery).

**Figure 7:**
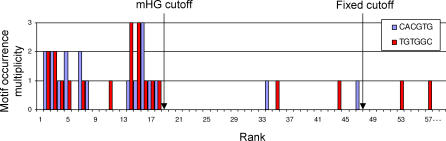
Motif Occurrences in the Top 59 (of ∼6,000) Promoters That Were Ranked According to Met32 Binding Signal A comparison is made between the data-driven mHG cutoff and the arbitrary fixed cutoff. It can be seen that the motifs are significantly more enriched when the list is partitioned using the mHG cutoff.

To address challenge (c1), instead of choosing an arbitrary cutoff for set partition, we search for a cutoff that partitions the data in a way that maximizes the motif enrichment. We present evidence that shows that the flexible mHG cutoff outperforms the rigid cutoff. One example of this is shown in Figure 5, where the flexible cutoff yields better results for all the tested TFs. Another example of the advantage of a flexible cutoff is the two motifs detected in three TFs involved in the sulfur amino acid pathway (Met4, Met31, and Met32). Figure 7 shows the number of motif occurrences in each of the top 59 promoters that were ranked according to Met32 binding signal (data from [25]). The motifs are highly frequent in the top 18 promoters, after which a strong drop in motif frequency is observed. DRIM identifies this, and partitions the set accordingly. In comparison, relying on the standard cutoff of 10^−3^ results in a target set of the top 48 promoters, most of which do not contain this motif. The signal-to-noise ratio is thus diminished, which may explain why these motifs were previously overlooked.

While the flexible cutoff is advantageous in many instances, it also introduces a multiple testing problem. To circumvent this (without resorting to strict multiple testing corrections that may mask the biological signal), we developed an efficient algorithm for computing the exact *p*-value of a given mHG score. This addresses challenge (c2). Another advantage of this exact statistical score is its straightforward biological interpretation: the mHG *p*-value reflects the probability of seeing the observed density of motif occurrences at the top of the ranked list under the null assumption that all configurations of motif occurrences are equiprobable.

Motif multiplicity is often indicative of biological function. It is therefore paramount to incorporate this type of information into the motif prediction model. We do so in a data-driven manner by developing the multi-mHG framework, thus addressing challenge (c3). The advantages of the multi-mHG model over the binary model are presented in Results, Binary versus multidimensional enrichment.

False prediction of motifs in randomly generated data is often mentioned as one of the drawbacks of computational motif discovery [25]. We report the testing of DRIM on random permutations of ranked sequences. When tested on more than 100,000 motifs, DRIM did not identify any significant motifs, thus addressing challenge (c4). The low false-positive prediction of our method is mainly attributed to the fact that it is based on rigorous statistics and relies on an exact *p*-value.

Another important issue that still requires consideration is the characterization of the motif search space. In this study we performed an exhaustive scanning of a restricted motif space (containing ∼10^5^ motifs) followed by a heuristic search for larger motifs. However, the motif search space can be further extended to include motifs that are longer, “fuzzier,” or more complex. Additional considerations such as the distance of the motif from the transcription start site may be taken into account as well as logical relations between different motifs (e.g., “OR,” “AND” operations). It is clear that many of these features are required to correctly model complex regulation patterns that are observed in higher eukaryotes. Two inherent limitations need to be considered when extending the search space: first, as the size of the motif search space increases, the problem of efficiently searching the defined space becomes more acute in terms of running time. Second, since the size of the search space is virtually endless, the problem of multiple testing rapidly erodes the signal-to-noise ratio, requiring an appropriate refinement of the statistical models.

To test our method, we constructed a dataset containing ChIP–chip experiments of 203 putative TFs in Saccharomyces cerevisiae [25,28]. Surprisingly, we discovered a significant length bias in roughly one-third of these experiments. One possible explanation for this phenomenon is nonspecific binding between TFs and DNA, which causes longer sequences to bind more TFs. This explanation is also consistent with the “TF sliding hypothesis” [48]. Why only some TFs exhibit this length bias binding tendency remains an open question. To avoid false positives due to this phenomenon, we opted to filter out all ChIP–chip experiments that had significant length bias. Future work should address this point and focus on developing statistics that are insensitive to this type of bias.

We analyzed the filtered dataset using DRIM and report novel putative TFBS motifs. Additional evidence that indicates the newly discovered motifs are biologically functional was also presented. One interesting finding is that the Aro80 motif we identified, which exists only in seven copies throughout the entire yeast genome, resides in Aro80′s own promoter. This finding suggests that Aro80 regulates its own transcription by binding to its own promoter. Additionally, three GATA binding sites that reside in the Aro80 promoter adjacent to the motif occurrence lead us to speculate that Aro80′s putative self binding is inhibited by competing GATA binding factors (for details see Figure 4B).

Another interesting observation is the CA repeat motifs, which we identified in seven different yeast TFs as well as in human DNA methylation. This type of low complexity motifs have so far been mostly ignored or filtered out by other computational methods. By contrast there is no need to resort to this type of artificial filtering when using the mHG statistics. Our findings in yeast suggest that for certain TFs there is a significant correlation between a sequence's capacity to bind a TF and the presence of a CA repeat in the sequence. This supports a previous hypothesis that CA repeats alter the structure of DNA and thus contribute to TF binding [34]. Our findings constitute concrete evidence of this phenomenon and suggest it may be more frequent than previously appreciated.

We also applied DRIM to high-throughput measurements of methylated CpG islands [2] in human cancer cells, in order to try to identify motifs that are enriched in hypermethylated regions. Interestingly, we identified GA and CA repeat elements as highly enriched in methylated CpG regions of four different cancer cell lines. This is in agreement with previous studies of CpG methylated regions in other cell lines [39,40]. It is interesting to ask whether these repeat elements play some active role in CpG methylation. In [40] the authors give statistical argumentation against such a hypothesis. Instead, they hypothesize that CA (or TG) repeats are caused by an increased mutation rate of methylated CpGs that are deaminated into TpGs. Even if true, this still does not explain the enrichment of the GA repeats. Further experimental and bioinformatic interrogation of this point is therefore called upon.

Overall, DRIM discovered ten novel motifs in methylated CpG regions. Strikingly, nine of them are similar to DNA sequence elements that bind the Polycomb complex in *Drosophila* and/or human [41,42, 44]. The Polycomb complex is involved in epigenetic silencing via histone methylation. The suggested link between histone methylation and CpG methylation is in agreement with recent work that demonstrated the EZH2 protein interacts with DNA methyltransferases via the Polycomb complex [45]. We also note that the DNA sequence motifs of the two pathways were conserved in *Drosophila* and human, which is complementary to the observation that the Polycomb proteins are evolutionarily conserved [44,49]. Many of the motifs we found in the CpG methylation data are similar across different types of cancer cell lines. This may suggest that the CpG methylation mechanism is orchestrated by DNA binding factors that are similar in different types of cancer cell lines.

Perhaps the most important conclusion that can be drawn from this study is that looking at biological sequence data in a ranked manner rather than using an arbitrary fixed cutoff to partition the data enables the detection of biological signals that are otherwise overlooked. This suggests that other motif detection methods that rely on fixed cutoffs may benefit from dynamic partitioning. While the effectiveness of our approach was demonstrated on ChIP–chip and methylation data, it can also be applied to a wide range of other data types such as expression data or GO analysis. The DRIM application is publicly available at http://bioinfo.cs.technion.ac.il/drim.

## Materials and Methods

### The minimum hypergeometric score.

In this subsection we introduce the basics of the mHG statistics, and demonstrate how it can be applied in a straightforward manner to eliminate the need for an arbitrary choice of threshold. To explain the biological motivation of mHG, consider the following scenario: suppose we have a set of promoter regions each associated with a measurement, e.g., a TF binding signal as measured by ChIP–chip [1]. We wish to determine whether a particular motif specified in IUPAC notation, say CASGTGW, is likely to be a TFBS motif. We rank the promoters according to their binding signals—strong binding at the top of the list and the weak at the bottom (Figure 1i). Next, we generate a binary occurrence vector with one or zero entries dependent on whether or not the respective promoter contains a copy of the motif (Figure 1ii). For simplicity we ignore cases where a promoter contains multiple copies of the motif (a refined model, which takes motif multiplicity into account, will be discussed later). Motifs that yield binary vectors with a high density of 1′s at the top of the list are good candidates for being TFBS.

Let us assume for the moment that we know the correct physical-based cutoff on the TF binding signal. The data could then be separated into “strong binding promoters” (i.e., the target set) and “weak binding promoters” (i.e., the background set). We are now interested to know whether there is a particular motif for which the target set contains significantly more motif occurrences than the background set. Let *N* be the total number of promoters *B* of which contain the motif, and *n* the size of the target set. Let *X* be a random variable describing the number of motif occurrences in the target set. Assuming a uniform distribution over all occurrence vectors with these characteristics, the probability of finding *exactly b* occurrences in the target set has a hypergeometric distribution, namely:

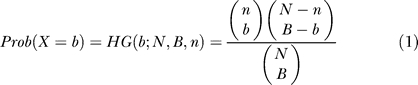
The tail probability of finding *b or more* occurrences in the target set is:

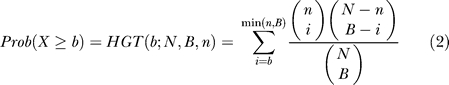



As we don't really always have a strict definition of the target set, we employ a strategy that seeks a partition for which the motif enrichment is the most significant, and compute the enrichment under that particular partition. Formally, consider a set of ranked elements and some binary labeling of the set λ = *λ*_1_,…,*λ_N_* ∈ {0,1}*^N^*. The binary labels represent the attribute (e.g., motif occurrence). The mHG score is defined as:

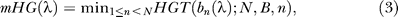
where *b_n_*(λ) = 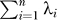 . In words, the mHG score reflects the surprise of seeing the observed density of 1's at the top of the list under the null assumption that all configurations of 1's in the vector are equiprobable. The cutoff between the top of the list and the rest of the list is chosen in a data-driven manner so as to maximize the enrichment (Figure 1iii). We discuss other variants of the mHG score in Texts S2 and S3.

### Calculating the *p*-value of the mHG score.

The mHG flexible choice of cutoff introduces a multiple testing complication and therefore gives rise to the need for computing the exact *p*-value. In Text S1 and Figure S2 we demonstrate several bounds for mHG *p*-values. These bounds may be used for rapid assessment of the *p*-value of a given mHG score, which can be instrumental in improving algorithmic efficiency. In this section, we describe a novel dynamic programming algorithm for calculating the exact *p*-value of a given mHG score. This approach is related to a previously described approach for calculating exact *p*-values of other combinatorial scores ([50,51], with details in [52]).

**Figure 8:**
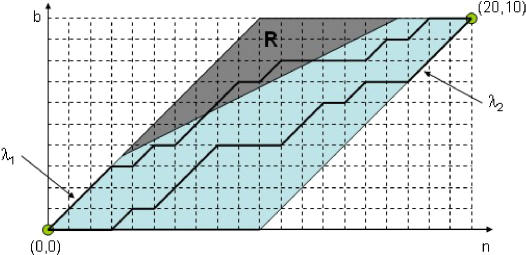
Two-Dimensional Grid Used for Calculating mHG *p*-Value In this example *N* = 20, *B* = 10, *p* = 0.1. Light-shaded area describes all attainable values of *n* and *b*. Dark-shaded area describes the subset *R*: all values of *n* and *b* for which HGT(b;N,B,n) ≤ *p*. Two (0,0) → (*N*,*B*) paths are depicted, representing the binary label vectors *λ*_1_ = {1,1,1,0,1,0,1,1,1,0,1,0,.0,0,0,1,0,1,0,0} and *λ*_2_ = {0,0,0,1,0,1,1,1,0,0,0,1,1,0,1,0,0,1,1,1}. The path λ_1_ traverses *R,* demonstrating that *mH*G (*λ*_1_) ≤ *p*. The path λ_2_ does not traverse *R,* demonstrating that *mH*G (*λ*_1_) > *p*.

As noted in the previous section, the mHG score depends solely on the content of the label vector *λ*. Set *N* and *B,* and consider the space of all binary label vectors with *B* 1′s and *N*−*B* 0′s: Λ = {0,1}^(*N−B*,*B*)^. Assume that we are given a vector *λ*_0_∈Λ, for which we calculate the mHG score *mHG*(*λ*_0_) = *p*. We would like to determine pval(p) = Prob(mHG(*λ*) ≤ p) under a uniform distribution of vectors in Λ. Given an mHG score *p,* we do this by means of path counting. The space of all label vectors Λ = {0,1}^(*N−B*,*B*)^ is represented as a two-dimensional grid ranging from (0,0) at the bottom left to (*N*,*B*) at the top right. Each specific label vector *λ*∈Λ is represented by a path (0,0) → (*N*,*B*) composed of *N* distinct steps. The *i*th step in the path describing a vector *λ* is (1,0) if λ*_i_* = 0 and (1,1) if λ*_i_* = 1 (see Figure 8). Each point (*n*,*b*) on the grid corresponds to a threshold (on ranks) *n,* and the respective value *b* = *b_n_*(1). It can therefore be associated with a specific HGT score: *HGT_n_*(*λ*) = *HGT* (*b_n_*(*λ* );*N*,*B*,*n*). A subset of the points on the grid can be characterized as those points (*n*,*b*) for which *HGT* (*b*;*N*,*B*,*n*) ≤ *p*. We denote this subset *R* = *R*(*p*) (see Figure 8).

The (0,0) → (*N*,*B*) path representing λ visits *N* distinct grid points (excluding the point (0,0)), representing the *N* different HGT scores that are considered when calculating its mHG score: *mHG*(*λ*) = min_1≤*n*<*N*_ *HGT_n_*(*λ*). *mHG*(*λ*) ≤ *p* if the path representing *λ* visits *R*. Denote by Π(*n*,*b*) the total number of paths (0,0) → (*n*,*b*) and by Π*_R_*(*n*,*b*) the number of paths (0,0) →(*n*,*b*) not visiting *R*. We then have:

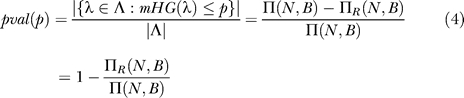



We calculate Π*_R_*(*n*,*b*) by means of dynamic programming. Initially, set Π*_R_* (0,0) = 1 and Π*_R_*(*n*,*b*) = 0 for *b* = −1 and along the diagonal *b* = *n* + 1, 0 ≤ *n* ≤ *B*. Then, for each 1 ≤ *n* ≤ *N*, and max(0,*B* − *N* + *n*) ≤ b ≤ min(*B*,*n*) calculate Π*_R_*(*n*,*b*) using the formula:

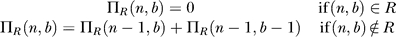



In total, we perform a O(*N* ^2^) routine in order to calculate Π*_R_*(*N*,*B*) for a given score *p*. Trivially, we have Π(*N, B*) = 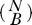 and pval(*p*) may be directly computed from Equation 4.

### Multidimensional mHG score.

So far we have dealt with enrichment of binary attributes, in which a one or zero indicated whether or not the attribute appeared. There are cases where one would like to associate a number with an attribute. We revisit the scenario we described in previous sections in which we tried to determine whether a particular motif is likely to be a TFBS motif. The promoters were ranked according to their binding signals, and the corresponding binary occurrence vector was generated. Notice that some promoters may contain several copies of a particular motif. Clearly, this information is valuable and should be incorporated in the enrichment analysis. How exactly to incorporate this information is not clear. For example, consider two motif occurrence vectors generated for two different motifs, where the top ten entries of the vectors are all 1's and all 2's, respectively. Is the second motif more enriched than the first? Clearly, this depends on the rarity of double motif occurrences compared with single occurrences in the corresponding vectors. If the frequency of 2's is lower than that of 1's, then the second motif is more significant. However, if they are equally frequent (this is often the case for degenerate motifs such as poly A's) then both motifs are equally enriched.

To quantitatively capture this notion and address motif multiplicity in a data-driven manner, we propose a multidimensional hypergeometric model, which extends the previously defined framework for enrichment analysis to nonbinary label vectors. Formally, let *λ* be a uniformly drawn label vector *λ* = *λ*_1_,…,*λ_N_* ∈ {0…*k*}*^N^* containing *B*_1_ 1's, *B*_2_ 2's … *B_k_* k's and 
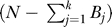
 . We would like to test for enrichment of 1's, 2's...k's at the top of *λ*. We define the multidimensional hypergeometric score (multiHG) for a set *S* of size *N* consisting of *k* + 1 subsets *S*_0_, *S*_1_, *S*_2_ … , *S*_k_ of respective sizes *N* – (*B*_1_ + *B*_2_ + …*B*_k_), *B*_1_, *B*_2_…, *B*_k_. Given a subset *S′ ⊂ S* of size *n,* the probability of finding exactly *b*_1_ elements of *S*_1_ and *b*_2_ elements of *S*_2_…, *b_k_* elements of *S_k_* within *S′* is:

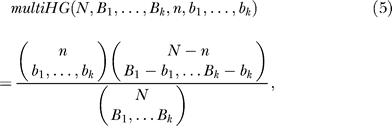
Let *X*_1_,…*X*_k_ be random variables describing the number of 1′s, … ,k's, respectively, at the top *n* positions of *λ*. The multihypergeometric tail probability (multiHGT) of seeing at least *b*_1_ 1's, at least *b*_2_ 2's,…, and at least *b_k_* k's at the top *n* positions of the vector is:

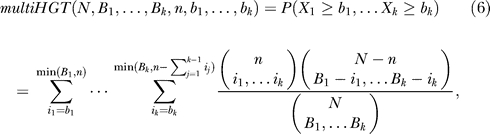



The definition of the mHG score can now be extended to the minimum of the set of multiHGTs calculated on all prefixes of *λ*.

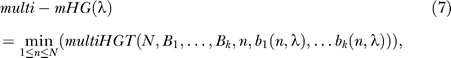
where *b_j_*(*n*, λ) =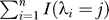 . Exact *p*-values for the multidimensional mHG, under a uniform null distribution, can be computed in a *k-*dimensional space using a path enumeration strategy similar to the one we used in the binary case. The details on how to compute this *p*-value in a three-dimensional space are explained in Text S4.

### The DRIM software.

The software tool DRIM implements the mHG framework for motif identification in ranked DNA sequences. A flow chart of DRIM is provided in Figure 1. In the rest of this section we describe the details of this implementation.

*Exhaustive search of the restricted motif space*. Ideally we would like to exhaustively search through the space of all biologically viable motifs and identify those that are significantly enriched at the top of the ranked list. However, this is infeasible in terms of running time (the space of viable TF binding sites includes motifs of size up to 20, i.e., 15^20^ k-mers). We therefore resort to a simple strategy where the motif search is broken into two stages: first an exhaustive search on a restricted motif space is performed. The “motif seeds” that are identified in the preliminary search are used as a starting point for a heuristic search of larger motifs in the entire motif space. The restricted motif space *S* used in this study is the union of two subspaces *S*_1_ and *S*_2_: *S*_1_ = {*A*,*C*,*G*,*T*,*R*,*W*,*Y*,*S*,*N*}^7^, where the IUPAC degenerate symbols (i.e., *R*,*Y*,*W*,*S*,*N*) are restricted to a maximum degeneracy of 2 and *S*_2_ = {*A*,*C*,*G*,*T*}^3^ *N* ^3−25^{*A*,*C*,*G*,*T*}^3^. The rationale behind the usage of the restricted IUPAC alphabet in *S*_1_ instead of the complete 15 symbol alphabet stems from DNA–TF physical interaction properties and TFBS database statistics as explained in previous work [53]. *S*_2_ captures motifs that contain a fixed gap (different motifs can have different gap sizes), which is characteristic of some TFs such as Zinc fingers).

**Figure 9:**
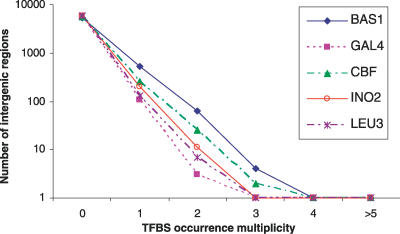
The Distribution of TFBS Occurrence Multiplicities per Intergenic Region in S. cerevisiae Is Shown for Five TFs Whose TFBS Motif Was Experimentally Verified Note that the *y*-axis is logarithmic. It can be seen that in most instances the TFBS appears in either zero, one, or two copies per intergenic region.

*mHG enrichment*. For each of the motifs in *S,* we generate a ranked occurrence vector and compute the enrichment in terms of the multidimensional mHG. Due to running time considerations, we restrict the multidimensional mHG to three dimensions. This means that the model assumes each intergenic region contains either 0, 1, or ≥2 copies of a motif. To test whether this assumption is reasonable in the case of true TFBS motifs, we examined the occurrence distribution of TFBS motifs that were experimentally verified in S. cerevisiae (see Figure 9). It can be seen that the assumption holds for the five TFs that were tested since the majority of all intergenic regions contained either zero, one, or two copies of the TFBS. At the end of this stage, only motif seeds with mHG score <10^−3^ are kept. Similar motifs are filtered (as explained in Texts S5 and S6), and the remaining motif seeds are fed into the heuristic search module for expansion, Figure 1iii–1iv.

*Motif expansion by heuristic search*. The filtered motif seeds are used as starting points for identifying larger motifs that do not reside in the restricted motif space. This is done through an iterative heuristic process that employs simulated annealing. The objective function is to minimize the motif mHG *p*-value. We tested two different strategies for determining valid moves in the motif space. In the first, we defined a transition from motif M1 to M2 as valid if M1 and M2 are within a predefined Hamming distance *D,* with all valid moves being equiprobable. Additional bases can also be added to the motif flanks, thus enabling motif expansion. Note that the mHG adaptive cutoff is recalculated at each step. In the second strategy, all the motif occurrences in the target set that are within Hamming distance *D* are aligned. A consensus motif above IUPAC is extracted and the algorithm attempts a transition to that motif. While the second strategy converges much faster than the first, it is also more prone to converge to local minima (in the final application we use the second strategy with *D* = 1). At the end of the process, the exact *p*-value of each of the expanded motifs is computed. To correct for multiple motif testing, the *p*-value is then multiplied by the motif space size. Only motifs with corrected *p*-value <10^−3^ are reported.

*Optimizations and running time*. The DRIM application was implemented in C++. A “blind search” requires ∼100,000 motifs to be checked for enrichment in each run. It is therefore paramount to optimize the above-described procedures to enable a feasible running time. There are two bottlenecks in terms of running time: the motif occurrence vector generation and the mHG computation. We developed several optimization schemes to improve both. In the final configuration, the running time on a list of 6,000 sequences with an average size of 480 bases took ∼3 minutes on a Pentium IV, 2 GHz.

### Characteristics of datasets.

*ChIP–chip dataset*. A number of assays have been recently developed that use immunopercipitation-based enrichment of cellular DNA for the purpose of identifying binding or other chemical events and the genomic locations at which they occur. Location analysis, also known as ChIP–chip, is a technique that enables the mapping of transcription binding events to genomic locations at which they occur [1,54]. The output of the assay is a fluorescence dye ratio at each spot of the array. If spots are taken to represent genomic regions, then we can regard the ratio and *p*-value associated with each spot as an indication of TF binding in the corresponding genomic region. We applied DRIM to S. cerevisiae genome-wide location data reported in Harbison et al. [25] and Lee et al. [28]. The first consists of the genomic occupancy of 203 putative TFs in rich media conditions (YPD). In addition, the genomic occupancy of 84 of these TFs was measured in at least one other condition (OC). In each of the experiments, the genomic sequences were ranked according to the TF binding *p*-value. Surprisingly, we observed that 69 of the 203 ranked sequence lists of YPD had significantly longer sequences at the top of the list (first 300 sequences) compared with the rest of the list with *t*-test *p*-value ≤ 10^−3^. We observed a similar phenomenon in 76 of the 148 ranked sequence lists of OC experiments (see Figure S1). In other words, for some TFs, *longer sequences are biased toward stronger binding signals*. This observation is unexpected since, although longer probes hybridize more labeled material than shorter probes, the increase should be proportional in both channels. This type of length bias may cause spurious results under our model assumptions and hence the final dataset, termed “Harbison filtered dataset,” refers to the remaining 207 experiments (135 YPD, and 72 OC) of 162 unique TFs that did not have length bias (Table S1).

An additional ChIP–chip dataset was constructed using the data reported in Lee et al. [28] containing 113 experiments in rich media. The data is partially exclusive to the data of Harbison et al. [25]. The same filtering procedure was performed, resulting in a set of 65 experiments, termed “Lee filtered dataset.”

*Methylated CpG dataset*. Using a technique similar to ChIP–chip, termed methyl-DNA immunoprecipitation (mDIP), enables the measurement of methylated CpG island patterns [2,55]. The third dataset contains the CpG island methylation patterns of four different human cancer cell lines (Caco-2, Polyp, Carcinoma, PC3) where several replicate experiments were done for each of the cell lines. In each of these experiments, the CpG methylation signal was measured in ∼13,000 gene promoters as reported in [2].

## Supporting Information

Figure S1 Observed versus Expected Length Bias For each of the 148 OC ChIP–chip experiments reported in [25], we ranked the yeast intergenic sequences according to their binding signal. The lengths of the top 300 sequences in each experiment were compared with the lengths of the rest of the sequences using a student *t*-test. The *x*-axis is the *t*-test *p*-value and y(x) is the number of TF experiments with p ≤ x. The blue line is the observed cumulative distribution of the *t*-test *p*-values in the 148 experiments. The red line is the expected cumulative distribution of *t*-test *p*-values in randomly permuted sequence rankings. It can be seen that more than half of the ChIP–chip experiments have a statistically significant difference between the lengths of sequences that bind the TF the strongest compared with the lengths of the rest of the sequences.
(21 KB PNG)

Figure S2 Comparison of *p-*Value Bounds, Exact *p*-Value Calculation, and Observed Frequencies of mHG Scores for Two Synthetic Cases: (A) N = 600, B = 300; (B) N = 330, B = 30 In each case the following values were generated for several different mHG scores: lower bound (p), trivial upper bound (Np), tighter upper bound (Bp), exact *p*-value calculation (pVal), and observed *p*-values over 10,000 random instances (sim). Note the improvement of the tighter upper bound (Bp) when N ≫ B.
(13 KB PNG)

Figure S3 Comparison of mHG Score and *p*-Value Distributions for Motifs in Randomly Ranked Sequences with Those of True TFBS Motifs in Ranked Lists Derived from the Corresponding ChIP–chip Assays ∼100,000 motifs were scanned in 400 randomly ranked genomic sequences, and their corresponding corrected *p*-value (A) and mHG score (B) were recorded. The corrected *p*-values involve two levels of multiple test corrections: correction on the number motifs that were tested; and correction for the multiple cutoffs that are tested as part of the mHG optimization process. None of the tested motifs had a corrected *p*-value < 10^−3^. DRIM was applied on the ChIP–chip data of five TFs and the mHG scores, and corrected *p*-values of the true TFBS motifs (as previously determined experimentally) were recorded. In all instances, the true TFBS motifs were predicted with *p*-values that were several orders of magnitude more significant than the best random set motif *p*-value.
(81 KB JPG)

Figure S4 Compatibility between the BS_Aro80_ Motif Identified by DRIM and Previously Reported Mutagenesis Studies [32] The Aro9 promoter region from base −169 to −133 as well as six other copies containing mutations and deletions are shown. These regions were used to construct hybrid promoters and measure the expression of a reporter gene, which is dependent on the binding of Aro80 to the promoter [32]. The two partially overlapping copies of BS_Aro80_ that reside in the Aro9 promoter and an additional sequence element that is similar to the canonic BS_Aro80_ (two different bases) are marked with green and blue arrows, respectively. It can be seen that the expression values are highly compatible with the number of intact BS_Aro80_ copies, i.e., more intact copies yield higher expression.
(11 KB PNG)

Figure S5 Met4-Met28-CBF and Met4-Met28-Met31/32 Complexes Binding to DNA (A) Schematic representation of Met4-Met28-CBF and Met4-Met28-Met31/32 complexes [37,38].
(B) A hypothetical Met4-Met28-CBF-Met31/32 complex. Immunoprecipitation of any of the TFs in the complex will precipitate the same set of sequences, which explains why DRIM identifies the same two motifs for all TFs in the complex.
(67 KB PNG)

Figure S6 Comparison of the mHG and HG Methods on Simulations of Motif Occurrence Vectors The vectors were generated according to a rank-dependent distribution (see the section, Comparing mHG and HG on simulated motif occurrences) with 18 different parameter combinations (a = 10, 50, 100; b = 0.01, 0.05, 0.1; u = 0.01, 0.1). The −log fraction between mHG and HG *p*-values in cases where the *p*-value of one of the methods was smaller than 10^−3^ are shown.
(117 KB JPG)

Table S1 List of TFs in the Harbison Filtered Dataset (27 KB XLS)

Table S2 Motif Predictions of DRIM on the Harbison Filtered Dataset (245 KB XLS)

Table S3 Comparison between the Predictions of DRIM and Those Reported in [25] (18 KB XLS)

Table S4 Comparison between mHG Flexible Cutoffs and 10^−3^ Fixed Cutoffs in Yeast ChIP–chip Data (15 KB XLS)

Table S5 Gene Accession Numbers The National Center for Biotechnology Information (NCBI) (http://www.ncbi.nlm.nih.gov) accession numbers for the genes discussed in the paper.
(18 KB XLS)

Text S1 Bounds for the mHG *p*-Value (137 KB DOC)

Text S2 Partition-Limited mHG Score (24 KB DOC)

Text S3 mHG and Expression (24 KB DOC)

Text S4 *p*-Value of the Three-Dimensional mHG Score (47 KB DOC)

Text S5 Motif Similarity (38 KB DOC)

Text S6 Filtering Similar Motifs (24 KB DOC)

Text S7 Comparing mHG and HG on Simulated Motif Occurrences (21 KB DOC)

### Accession Numbers

Accession numbers for the genes discussed in the paper are given in Table S5.

## Abbreviations

ChIP–chip: chromatin immuno-precipitation on a microarray
DRIM: discovery of rank imbalanced motifs
mDIP: methyl-DNA immunoprecipitation
mHG: minimal hypergeometric
OC: other condition
TF: transcription factor
TFBS: transcription factor binding sites
YPD: rich media condition
