# Supplementary material for: Discovering Motifs in Ranked Lists of DNA Sequences
Source: PLoS Comput Biol. 2007 Mar 23;3(3):e39. doi: 10.1371/journal.pcbi.0030039 (PMC1829477; doi:10.1371/journal.pcbi.0030039)
Supplement: Supplementary file 1 [file pcbi.0030039.sd001.doc]

## Text S1 Bounds for the mHG p-value

We demonstrate several bounds for pval(*p*). These bounds may be used for rapid assessment of the p-value of a given mHG score, which can be instrumental in improving algorithmic efficiency. In Figure Error: Reference source not found we compare the bounds with exact p-values as well as with observed frequencies, on randomly generated test cases.

For fixed values of *N* and *B* we define$HGT_{n}(\lambda)=HGT(b_{n}(\lambda);N,B,n)$. $HGT(x;N,B,n)$ is the complement of the cumulative distribution function of a hyper-geometric random variable. As such, for any value *p* we have $Prob(HGT_{n}(\lambda)\leq p)\leq p\text{ }$^[1]^. Set an attainable mHG score *p*. If $mHG(\lambda)=p$ then there is some rank n^*^ at which this score was attained. Clearly . For similar reasons $\left\{\lambda:mHG(\lambda)\leq p\right\}=\bigcup_{n=1}^{N} \left\{\lambda:HGT_{n}(\lambda)\leq p\right\}$. This leads to the following (trivial) bounds for any $\text{ }HGT_{n}(\lambda)$ attainable *p*:

$p\leq Prob(mHG(\lambda)\leq p)\leq Np.$ ^5^	(8)

In many cases the upper bound provided in (8) is very loose. When *B*<<*N* the following tighter upper bound is of greater use (see Figure Error: Reference source not found). Fix *N* and *B*, and consider the space of all binary label vectors with *B* 1’s and *N*-*B* 0’s: $\Lambda=\{0,1\}^{(N-B,B)}$, endowed with a uniform probability measure.

**Theorem 1.1** *For any* $\text{ }HGT_{n}(\lambda)$ *attainable p:*

$p\leq Prob(mHG(\lambda)\leq p)\leq Bp.$ *(**9)*

##### Proof:

Note that we only need to test *B* thresholds to compute mHG(λ). A union bound may therefore seem applicable. However, since the thresholds depend on the point  and may be different for different s we need to use a more careful argument.

For each $1\leq i\leq B$ let be the maximal rank *n* for which any vector  with $b_{n}(\lambda)\geq i$ has a score $HGT_{n}(\lambda)\leq p$. Formally:

$n_{i}=max\left\{n:(b_{n}(\lambda)\geq i)\Rightarrow HGT_{n}(\lambda)\leq p\right\}.$ (10)

Monotonicity properties of the hyper-geometric distribution imply the existence of such numbers . By definition they are constants, independent of .

Any vector  for which $mHG(\lambda)\leq p$ attains this score at some rank $n^{*}=n^{*}(\lambda)\text{ }$(i.e. $mHG(\lambda)=HGT_{n^{*}}(\lambda)$). Let $\text{ }b^{*}=b_{n^{*}}(\lambda)$. By definition (10) $n_{b^{*}}\geq n^{*}\text{ }$and therefore $b_{n_{b^{*}}}(\lambda)\geq b_{n^{*}}(\lambda)=b^{*}\text{ }$ (the left hand side represents a larger prefix of the vector). Therefore, again from definition (10): $HGT_{n_{b^{*}}}(\lambda)\leq p$.

The above shows, in other words, that for any vector  for which $mHG(\lambda)\leq p$ there is some value $1\leq i\leq B$ (namely ) for which $\text{ }HGT_{n_{i}}(\lambda)\leq p$. Note that even though this *i* depends on  there are only *B* possible values and only *B* numbers . Therefore:

$\left\{\lambda:mHG(\lambda)\leq p\right\}\subseteq\bigcup_{i=1}^{B} \left\{\lambda:HGT_{n_{i}}(\lambda)\leq p\right\},$ (11)

and we can now apply a union bound to conclude:

$Prob(mHG(\lambda)\leq p)\leq Bp.$ (12)

1. If *X* and *Z* are discrete random variables that take a finite number of values and with probabilities and respectively, and *P*(*Z*=*x*)=*P*(*X**x*) then the following properties hold: (1)  p *P*(*Z**p*)*p*; (2) for any attainable *P*(*Z**p*)=*p*.

   ^5^
