# Supplementary material for: Discovering Motifs in Ranked Lists of DNA Sequences
Source: PLoS Comput Biol. 2007 Mar 23;3(3):e39. doi: 10.1371/journal.pcbi.0030039 (PMC1829477; doi:10.1371/journal.pcbi.0030039)
Supplement: Supplementary file 2 [file pcbi.0030039.sd002.doc]

## Text S2 Partition-limited mHG score

Recall that mHG is as the minimum HGT score calculated over all partitions that agree with the given ranking. In many cases it is reasonable to consider HGT scores obtained only at a subset of all possible partitions, either due to some external information, or for algorithmic efficiency. Two possible partition-limited variations are:

•	Consider only the first *n_max_* partitions. This is useful when we are interested mainly in enrichment that occurs at the top of the rank list, and not at the bottom.

•	Consider only every *n_step_*th partition. This is useful for improving algorithmic efficiency and tightening bounds, at the expense of the accuracy of the mHG score.

For both of these mHG variations, it is possible to devise appropriate bounds and algorithms for computing the accurate p-value.
