# Supplementary material for: Discovering Motifs in Ranked Lists of DNA Sequences
Source: PLoS Comput Biol. 2007 Mar 23;3(3):e39. doi: 10.1371/journal.pcbi.0030039 (PMC1829477; doi:10.1371/journal.pcbi.0030039)
Supplement: Supplementary file 3 [file pcbi.0030039.sd003.doc]

## Text S3 mHG and expression

The mHG score can also be applied to expression data. Specifically, different rankings of sequences may be used to interrogate different hypotheses. Some straightforward examples include:

•	Ranking according to expression level - over-expressed genes at the top.

•	Ranking according to expression level - under-expressed genes at the top.

•	Ranking according to differential expression.

In some cases one may be interested in computing HGT scores of sets that contain elements from both the top and the bottom of a ranked list. For example, some TFs such as Rap1, may bind to promoters and cause either over or under-expression. In such cases it is reasonable to search for motifs that are enriched in a target set containing the promoters of the over-expressed and under-expressed genes. Instead of using two fixed thresholds on the expression signal, one for determining over and the other for under-expression, it is possible to use the following variant of mHG: Consider all partitions of the data in which the target set contains the top *n* and the bottom *m* sequences and compute the mHG score over these partitions.
