# Supplementary material for: Discovering Motifs in Ranked Lists of DNA Sequences
Source: PLoS Comput Biol. 2007 Mar 23;3(3):e39. doi: 10.1371/journal.pcbi.0030039 (PMC1829477; doi:10.1371/journal.pcbi.0030039)
Supplement: Supplementary file 4 [file pcbi.0030039.sd004.doc]

## Text S4 P-value of the 3-dimensional mHG score

For fixed values of$N,B_{1},B_{2}$, the p-value of a given 3-dimensional score *p* is calculated using a 3-dimensional grid ranging from (0, 0, 0) to$\text{(}N,B_{1},B_{2})$. A specific trinary label vector  is represented by a path $(0,0,0)\to(N,B_{1},B_{2})$where the *i*th step in the path is (1,0,0) if , (1,1,0) if or (1,0,1) if . Each point $(n,b_{1},b_{2})$ on the grid corresponds to a rank *n* and the respective values $\text{ }b_{k}=b_{k,n}(\lambda)$, and can be associated with a specific $HGT^{(3)}$score: $HGT_{n}^{(3)}(\lambda)=HGT(b_{1,n}(\lambda),b_{1,n}(\lambda);N,B_{1},B_{2},n)$ . As in the binary case, we define *R* to be the subset of the points on the grid for which $HGT_{n}^{(3)}(\lambda)\leq p$, and count the number of paths $\text{ }(0,0,0)\to(N,B_{1},B_{2})$ not traversing *R* by dynamic programming, albeit at .
