# Supplementary material for: Discovering Motifs in Ranked Lists of DNA Sequences
Source: PLoS Comput Biol. 2007 Mar 23;3(3):e39. doi: 10.1371/journal.pcbi.0030039 (PMC1829477; doi:10.1371/journal.pcbi.0030039)
Supplement: Supplementary file 5 [file pcbi.0030039.sd005.doc]

## Text S5 Motif similarity

We use the motif similarity measure in order to compare our predicted motifs to those reported in other work as well as when filtering similar motifs. First, we define a similarity measure between IUPAC symbols. Let α and β be symbols over the IUPAC alphabet (α, {*A*, *C*, *G*, *T*, *R*, *Y*, *W*, *S*, *M*, *K*, *H*, *B*, *V*, *D*, *N*}). Each IUPAC symbol can also be represented by the corresponding subset of {*A*, *C*, *G*, *T*}. We define a distance between α and β as follows:

$\delta(\alpha,\beta)=1-2\frac{|\alpha\cap\beta|}{|\alpha|+|\beta|}.$ (13)

Notice that this metric maintains the following properties:

1.	For identical symbols (α,β)=0 (i.e. identical symbols have a distance of 0) ;

2.	For disjoint symbols (α,β)=1 (e.g. {*A*} vs. {*C*} or *W*={*A*,*T*} vs. *S*={*C*,*G*}) ;

3.	$0\leq\delta\text{( }\alpha\text{, }\beta\text{) }\leq1$ (e.g. α={*A*,*C*,*G*}, β={*A*,*G*} (α,β)=0.2) .

We define the distance between two motifs *a* and *b* above IUPAC with sizes *m* and *n* as follows:

$D(a,b)=\int_{i=1}^{min(n,m)} \delta(a_{i},b_{i})+w_{1}u,$ (14)

where *u* is the number of unpaired bases and is the weight assigned to such occurrences. It is sometimes useful to allow shifts in the motif comparison. For example, *D*(*ACGTAC*, *CGTACG*) = 6, indicating that the two motifs are non-similar. However, a more careful examination reveals that the two motifs share a common substring containing 5 bases. To capture this type of similarity in the motif comparison we consider all *n*+*m*-1 possible shifts when aligning the motifs (no gaps are permitted in the alignment) and choose the one that minimizes the distance. When comparing our predicted motifs to those reported in the literature as well as when filtering similar motifs produced by our method we use = 0.6. Two motifs are considered similar if:

$\frac{D(a,b)}{mean(|a|,|b|)}\leq0.5.$ (15)
