# Supplementary material for: Discovering Motifs in Ranked Lists of DNA Sequences
Source: PLoS Comput Biol. 2007 Mar 23;3(3):e39. doi: 10.1371/journal.pcbi.0030039 (PMC1829477; doi:10.1371/journal.pcbi.0030039)
Supplement: Supplementary file 6 [file pcbi.0030039.sd006.doc]

## Text S6 Filtering similar motifs

The exhaustive search module produces a list of significant motifs seeds, many of which are highly similar and overlapping. In order generate a list of unique motifs we rank the motifs according to their enrichment and apply the following simple procedure:

(i)	Chose the most significant motif in the list.

(ii)	Erase all the motifs that are similar to it (motif similarity is defined in Error: Reference source not found)

(iii)	Repeat this process until list is empty.

The motifs that were chosen are used as input to the heuristic module.
