# Supplementary material for: Discovering Motifs in Ranked Lists of DNA Sequences
Source: PLoS Comput Biol. 2007 Mar 23;3(3):e39. doi: 10.1371/journal.pcbi.0030039 (PMC1829477; doi:10.1371/journal.pcbi.0030039)
Supplement: Supplementary file 7 [file pcbi.0030039.sd007.doc]

## Text S7 Comparing mHG and HG on simulated motif occurrences

To further assess the properties of mHG compared to fixed set partition HG we performed a series of simulations in which the different methods were tested on random motif occurrence vectors, each generated according to a predefined distribution. When generating the occurrence vectors we tried to mimic occurrence vectors that arise from biological data. We define the probability of observing a motif occurrence at rank *r* in a vector as:

$P=u+e^{-(a+r)b}.$ (16)

The parameter *u* is the probability of observing a motif independent of its position in the vector. This attempts to capture the background genomic noise. The exponent component in the equation decays as the vector rank increases. The parameters *a* and *b* control the rank-imbalance degree of the motif. For example, if *b* is fixed, the larger *a* the weaker the motif signal is.

We generated vectors with different parameter combinations. For each vector we computed the mHG p-value, and the HG p-value using the top 10, 100 and 1000 positions in the vector as a target set. The fraction between the mHG and the best HG p-value was recorded. Results are summarized in Figure Error: Reference source not found. It can be see that in most cases the mHG p-value is equal or better than that of the HG.
